# The Dengue or Break-Bone

**Published:** 1861-01

**Authors:** 


### *The Dengue or Break-Bone.*

This fever prevailed the last season in Savannah and other Southern cities. It was not, however, in our opinion, as *severe or general* as it was some years past. It might be called an epidemic, perhaps, with propriety, but we are satisfied, from our own observation, that many cases of *intermittent* and *remittent* fevers were styled *break-bone*, because of an existing white tongue, and an unusual amount of pain in the limbs. Dengue is a fever of one paroxysm. The most of the cases treated by us, though accompanied with neuralgic pains, were distinctly remittent or intermittent, and late in the fall almost every case lasted from three to six days, a large majority unattended with an eruption. The cases of intermittent had same character of tongue, and pain in the limbs, with characteristic intermission, and chills followed by fever. This is never the case in Dengue. Hence we are forced to the conclusion that these cases were intermittent and remittent fevers, modified by the epidemic tendency to break-bone—not genuine cases of the latter. We must state that we searched diligently for the eruption in our hospital and private practice. The eruption was found in comparatively few cases. The latter is not a constant accompaniment of Dengue, we admit, but its absence in so many cases, is at least corroborative evidence that the fever was not as general as some might be inclined to believe.—

Wherever there exists an epidemic tendency, we are prone to class diseases accordingly. When epidemic cholera prevails, every diarrhœa is called cholera—when yellow fever, every fever is treated as such, &c. More discrimination is required if we desire to arrive at truth, and obtain reliable statistical data.

It is a notorious game quacks play, by stating to the public that they have so many cases of yellow fever, or cholera, or diptheria, etc., and only lost so many, making the last incredably small. This is a plan, scientific perhaps, (?) of misrepresenting their success. In one case every fever, however simple, is put down as yellow—in the other, every intestinal disorder is numbered among the cholera cases—and all apthous affections classed as diptheria. The public is deceived and reputation gained. We hope that regular practitioners will be guarded in this particular, and never sacrifice truth to individual interest. There is another peculiarity of fevers in Savannah this fall, but we do not now propose to enlarge upon it, which is that as winter commenced and vicissitudes of temperature became greater, there was an evident tendency to *congestion*. This was manifest in all kinds of fevers—Remittent, Intermittent, and Break-Bone.—*Sav. Journal of Medicine.*

